# Scientometrics on interventions used for adherence of hypertension and diabetes therapies

**DOI:** 10.31744/einstein_journal/2020AO4723

**Published:** 2019-12-06

**Authors:** Julio de Souza Sá, Lucas França Garcia, Marcelo Picinin Bernuci, Mirian Ueda Yamaguchi

**Affiliations:** 1 Centro Universitário de Maringá, Maringá, PR, Brazil.

**Keywords:** Health promotion, Drug therapy, Hypertension, Diabetes mellitus, Community health nursing, Ambulatory care, Patient care team, Biomedical technology, Bibliometrics

## Abstract

**Objective:**

To identify interventions aimed to improve adherence to medical and non-medical antihypertensive and antidiabetic therapy.

**Methods:**

Scientometric study conducted in February and March 2018, based on data collected on PubMed ^®^ and SciELO databases, using the following search terms: “interventions to improve adherence to diabetes therapy”, “interventions to improve adherence to hypertension therapy” and “interventions to improve adherence to therapy for hypertension and diabetes”.

**Results:**

A total of 95 articles were selected. Scientific production increased as of 2009, with a higher number of studies published between 2015 and 2017. Most interventions described in literature were aimed at diabetic patients (46.31%). Face-to-face interventions were more common (46.31%), followed by telephone-based (31.58%) and digital (26.31%) interventions. North America stood out as the continent with the highest number of publications (68.42%), followed by Europe (14.74%). Most studies (63.16%) were based on a single type of intervention.

**Conclusion:**

Traditional intervention methods were more widely used to promote adherence to antihypertensive and antidiabetic therapy; digital technology emerged as a trend in interventions aimed to improve hypertension and diabetes-related health behaviors.

## INTRODUCTION

Hypertension and *diabetes mellitus* are among the major causes of death due to non-communicable chronic diseases (NCDs) worldwide, with a global prevalence of 22% in hypertensive individuals, and approximately 9.4 million related deaths per year.^1^ Global prevalence in diabetic individuals is roughly 9%, with more than 1.5 million related deaths annually.^2^ In these settings, adherence to medical and non-medical treatment of hypertension and diabetes is a major challenge in health promotion.^3^

The rate of hypertension- and diabetes-related complications is constantly on the rise in middle- and low-income countries. This is partly due to population aging and the need to adopt a healthy lifestyle.^1^ Lack of adherence to treatment is a major public health concern, with half of patients failing to comply with medical prescriptions.^4^ Antihypertensive and antidiabetic drugs are recommended only when non-medical interventions, such as dietary management, regular physical activity, and other practices associated with a healthy lifestyle have failed.^5^

Measures adopted to tackle NCDs have defined policies and actions worldwide.^6^ Studies investigating adherence to treatment have revealed pathways for the development of innovative strategies and behavioral interventions, aimed to support proper monitoring of prescribed therapies, with improved quality of life for patients.^4^

Interventions consist of health promotion actions leading to individual or collective behavior changes, according to the social context in which each individual is inserted, with a view to improving adherence to NCD therapy.^7^ A wide array of interventions can assist healthcare managers and services in NCD control, ranging from traditional methods, such as home visits and individual or group counselling, to technology-based approaches employed to send reminders or deliver content associated with health promotion.^8^

## OBJECTIVE

To describe the state-of-the-art of scientific publications related to the development of interventions aimed to improve adherence to antihypertensive and antidiabetic therapy.

## METHODS

Scienciometric study based on data collected between February and March 2018 via search of PubMed^®^ (https://www.ncbi.nlm.nih.gov/pubmed/) and Scientific Electronic Library Online (SciELO; https://www.scielo.org/) databases.

PubMed^®^ database search was conducted using the following search terms: “interventions to improve adherence to diabetes therapy”, “interventions to improve adherence to hypertension therapy” and “interventions to improve adherence to therapy for hypertension and diabetes”. SciELO database was searched using the following search terms: “ *intervenções para melhorar a adesão à terapia do diabetes* ”, “ *intervenções para melhorar a adesão à terapia de hipertensão* ” and “ *intervenções para melhorar a adesão à terapia da hipertensão e diabetes* ”.

Literature review and systematic literature review articles were excluded, and only original articles were selected for increased precision. Titles and abstracts were analyzed, and the articles classified under seven headings according to intervention type, as follows: face-to-face intervention, telephone-based intervention, digital intervention, indirect intervention, health education intervention, postal intervention or financial incentive intervention.

Data were entered into spreadsheets (Excel 2016) and tabulated according to year of publication, type of disease or target-audience, type of intervention, number of publications per continent and number of interventions per study.

## RESULTS

Database search using selected terms yielded 600 publications in PubMed^®^ and none in SciELO, between 2000 and 2018. Of these, 95 articles were selected following title and abstract analysis ( Table 1 - Appendix 1). Articles failing to meet inclusion criteria (505) were excluded.

**Table 1:** Articles included in the study

Authors	Year	Títle	Journal
Monroe AK, Pena JS, Moore RD, Riekert KA, Eakin MN, Kripalani S, Chander G	2018	Randomized controlled trial of a pictorial aid intervention for medication adherence among HIV-positive patients with comorbid diabetes or hypertension	AIDS Care. 2018;30(2):199-206
do Valle Nascimento TM, Resnicow K, Nery M, Brentani A, Kaselitz E, Agrawal P, Mand S, Heisler M	2017	A pilot study of a Community Health Agent-led type 2 diabetes self-management program using Motivational Interviewing-based approaches in a public primary care center in São Paulo, Brazil	BMC Health Serv Res. 2017;17(1):32
Wong CA, Miller VA, Murphy K, Small D, Ford CA, Willi SM, Feingold J, Morris A, Ha YP, Zhu J, Wang W, Patel MS	2017	Effect of Financial Incentives on Glucose Monitoring Adherence and Glycemic Control Among Adolescents and Young Adults With Type 1 Diabetes: A Randomized Clinical Trial	JAMA Pediatr. 2017;171(12):1176-83
Frias J, Virdi N, Raja P, Kim Y, Savage G, Osterberg L	2017	Effectiveness of Digital Medicines to Improve Clinical Outcomes in Patients with Uncontrolled Hypertension and Type 2 Diabetes: Prospective, Open-Label, Cluster-Randomized Pilot Clinical Trial	J Med Internet Res. 2017;19(7):e246
Davis SA, Carpenter D, Cummings DM, Lee C, Blalock SJ, Scott JE, Rodebaugh L, Ferreri SP, Sleath B	2017	Patient adoption of an internet based diabetes medication tool to improve adherence: a pilot study	Patient Educ Couns. 2017;100(1):174-8
Floyd BD, Block JM, Buckingham BB, Ly T, Foster N, Wright R, Mueller CL, Hood KK, Shah AC	2017	Stabilization of glycemic control and improved quality of life using a shared medical appointment model in adolescents with type 1 diabetes in suboptimal control	Pediatr Diabetes. 2017;18(3):204-12
Lewey J, Wei W, Lauffenburger JC, Makanji S, Chant A, DiGeronimo J, Nanchanatt G, Jan S, Choudhry NK	2017	Targeted Adherence Intervention to Reach Glycemic Control with Insulin Therapy for patients with Diabetes (TARGIT-Diabetes): rationale and design of a pragmatic randomised clinical trial	BMJ Open. 2017;7(10):e016551
Di Bartolo P, Nicolucci A, Cherubini V, Iafusco D, Scardapane M, Rossi MC	2017	Young patients with type 1 diabetes poorly controlled and poorly compliant with self-monitoring of blood glucose: can technology help? Results of the i-NewTrend randomized clinical trial	Acta Diabetol. 2017;54(4):393-402
Albini F, Xiaoqiu Liu, Torlasco C, Soranna D, Faini A, Ciminaghi R, Celsi A, Benedetti M, Zambon A, di Rienzo M, Parati G	2016	An ICT and mobile health integrated approach to optimize patients’ education on hypertension and its management by physicians: The Patients Optimal Strategy of Treatment(POST) pilot study	Conf Proc IEEE Eng Med Biol Soc. 2016;2016:517-20
Nelson LA, Mulvaney SA, Gebretsadik T, Ho YX, Johnson KB, Osborn CY	2016	Disparities in the use of a mHealth medication adherence promotion intervention for low-income adults with type 2 diabetes	J Am Med Inform Assoc. 2016;23(1):12-8
Vissenberg C, Stronks K, Nijpels G, Uitewaal PJ, Middelkoop BJ, Kohinor MJ, Hartman MA, Nierkens V	2016	Impact of a social network-based intervention promoting diabetes self-management in socioeconomically deprived patients: a qualitative evaluation of the intervention strategies	BMJ Open. 2016;6(4):e010254
Choudhry NK, Isaac T, Lauffenburger JC, Gopalakrishnan C, Khan NF, Lee M, Vachon A, Iliadis TL, Hollands W, Doheny S, Elman S, Kraft JM, Naseem S, Gagne JJ, Jackevicius CA, Fischer MA, Solomon DH, Sequist TD	2016	Rationale and design of the Study of a Tele-pharmacy Intervention for Chronic diseases to Improve Treatment adherence (STIC2IT): A cluster-randomized pragmatic trial	Am Heart J. 2016;180:90-7
Piette JD, Marinec N, Janda K, Morgan E, Schantz K, Yujra AC, Pinto B, Soto JM, Janevic M, Aikens JE	2016	Structured Caregiver Feedback Enhances Engagement and Impact of Mobile Health Support: A Randomized Trial in a Lower-Middle-Income Country	Telemed J E Health. 2016;22(4):261-8
Lynch CP, Williams JS, J Ruggiero K, G Knapp R, Egede LE	2016	Tablet-Aided BehavioraL intervention EffecT on Self-management skills (TABLETS) for Diabetes	Trials. 2016;17:157
Kravetz JD, Walsh RF	2016	Team-Based Hypertension Management to Improve Blood Pressure Control	J Prim Care Community Health. 2016;7(4):272-5
Mayberry LS, Berg CA, Harper KJ, Osborn CY	2016	The Design, Usability, and Feasibility of a Family-Focused Diabetes Self-Care Support mHealth Intervention for Diverse, Low-Income Adults with Type 2 Diabetes	J Diabetes Res. 2016;2016:7586385
Lin TY, Chen CY, Huang YT, Ting MK, Huang JC, Hsu KH	2016	The effectiveness of a pay for performance program on diabetes care in Taiwan: a nationwide population-based longitudinal study	Health Policy. 2016;120(11):1313-21
Reese PP, Kessler JB, Doshi JA, Friedman J, Mussell AS, Carney C, Zhu J, Wang W, Troxel A, Young P, Lawnicki V, Rajpathak S, Volpp K	2016	Two Randomized Controlled Pilot Trials of Social Forces to Improve Statin Adherence among Patients with Diabetes	J Gen Intern Med. 2016;31(4):402-10
Duke DC, Wagner DV, Ulrich J, Freeman KA, Harris MA	2016	Videoconferencing for Teens With Diabetes: Family Matters	J Diabetes Sci Technol. 2016;10(4):816-23
Schoenthaler A, De La Calle F, Barrios-Barrios M, Garcia A, Pitaro M, Lum A, Rosal M	2015	A practice-based randomized controlled trial to improve medication adherence among Latinos with hypertension: study protocol for a randomized controlled trial	Trials. 2015;16:290
Volpp KG, Troxel AB, Long JA, Ibrahim SA, Appleby D, Smith JO, Jaskowiak J, Helweg-Larsen M, Doshi JA, Kimmel SE	2015	A randomized controlled trial of co-payment elimination: the CHORD trial. [ClinicalTrials.gov NCT00133068].	Am J Manag Care. 2015;21(8):e455-64
Volpp KG, Troxel AB, Long JA, Ibrahim SA, Appleby D, Smith JO, Jaskowiak J, Helweg-Larsen M, Doshi JA, Kimmel SE	2015	A randomized controlled trial of negative co-payments: the CHORD trial	Am J Manag Care. 2015;21(8):e465-73
Fischer MA, Jones JB, Wright E, Van Loan RP, Xie J, Gallagher L, Wurst AM, Shrank WH	2015	A randomized telephone intervention trial to reduce primary medication nonadherence	J Manag Care Spec Pharm. 2015;21(2):124-31
Margolis KL, Asche SE, Bergdall AR, Dehmer SP, Maciosek MV, Nyboer RA, O’Connor PJ, Pawloski PA, Sperl-Hillen JM, Trower NK, Tucker AD, Green BB	2015	A Successful Multifaceted Trial to Improve Hypertension Control in Primary Care: Why Did it Work?	J Gen Intern Med. 2015;30(11):1665-72
Weiss DM, Casten RJ, Leiby BE, Hark LA, Murchison AP, Johnson D, Stratford S, Henderer J, Rovner BW, Haller JA	2015	Effect of Behavioral Intervention on Dilated Fundus Examination Rates in Older African American Individuals With Diabetes Mellitus: a Randomized Clinical Trial	JAMA Ophthalmol. 2015;133(9):1005-12
Xin C, Xia Z, Jiang C, Lin M, Li G	2015	Effect of pharmaceutical care on medication adherence of patients newly prescribed insulin therapy: a randomized controlled study	Patient Prefer Adherence. 2015;9:797-802
Fortuna RJ, Nagel AK, Rose E, McCann R, Teeters JC, Quigley DD, Bisognano JD, Legette-Sobers S, Liu C, Rocco TA	2015	Effectiveness of a multidisciplinary intervention to improve hypertension control in an urban underserved practice	J Am Soc Hypertens. 2015;9(12):966-74
Friedberg JP, Rodriguez MA, Watsula ME, Lin I, Wylie-Rosett J, Allegrante JP, Lipsitz SR, Natarajan S	2015	Effectiveness of a tailored behavioral intervention to improve hypertension control: primary outcomes of a randomized controlled trial	Hypertension. 2015;65(2):440-6
Wayne N, Perez DF, Kaplan DM, Ritvo P	2015	Health Coaching Reduces HbA1c in Type 2 Diabetic Patients From a Lower-Socioeconomic Status Community: A Randomized Controlled Trial	J Med Internet Res. 2015;17(10):e224
Leon N, Surender R, Bobrow K, Muller J, Farmer A	2015	Improving treatment adherence for blood pressure lowering via mobile phone SMS-messages in South Africa: a qualitative evaluation of the SMS-text Adherence SuppoRt (StAR) trial	BMC Fam Pract. 2015;16:80
Johnson RM, Johnson T, Zimmerman SD, Marsh GM, Garcia-Dominic O	2015	Outcomes of a Seven Practice Pilot in a Pay-For-Performance (P4P)-Based Program in Pennsylvania	J Racial Ethn Health Disparities. 2015;2(1):139-48
Shane-McWhorter L, McAdam-Marx C, Lenert L, Petersen M, Woolsey S, Coursey JM, Whittaker TC, Hyer C, LaMarche D, Carroll P, Chuy L	2015	Pharmacist-provided diabetes management and education via a telemonitoring program	J Am Pharm Assoc (2003). 2015;55(5):516-26
Kjeldsen LJ, Bjerrum L, Dam P, Larsen BO, Rossing C, Søndergaard B, Herborg H	2015	Safe and effective use of medicines for patients with type 2 diabetes – A randomized controlled trial of two interventions delivered by local pharmacies	Res Social Adm Pharm. 2015;11(1):47-62
Chamany S, Walker EA, Schechter CB, Gonzalez JS, Davis NJ, Ortega FM, Carrasco J, Basch CE, Silver LD	2015	Telephone Intervention to Improve Diabetes Control: A Randomized Trial in the New York City A1c Registry	Am J Prev Med. 2015;49(6):832-41
Cassimatis M, Kavanagh DJ, Hills AP, Smith AC, Scuffham PA, Gericke C, Parham S	2015	The OnTrack Diabetes Web-Based Program for Type 2 Diabetes and Dysphoria Self-Management: a Randomized Controlled Trial Protocol	JMIR Res Protoc. 2015;4(3):e97
Baynouna LM, Neglekerke NJ, Ali HE, ZeinAlDeen SM, Al Ameri TA	2014	Audit of healthy lifestyle behaviors among patients with diabetes and hypertension attending ambulatory health care services in the United Arab Emirates	Glob Health Promot. 2014;21(4):44-51
Jaser SS, Patel N, Linsky R, Whittemore R	2014	Development of a positive psychology intervention to improve adherence in adolescents with type 1 diabetes	J Pediatr Health Care. 2014;28(6):478-85
Bobrow K, Brennan T, Springer D, Levitt NS, Rayner B, Namane M, Yu LM, Tarassenko L, Farmer A	2014	Efficacy of a text messaging (SMS) based intervention for adults with hypertension: protocol for the StAR (SMS Text-message Adherence suppoRt trial) randomised controlled trial	BMC Public Health. 2014;14:28
Leslie RS, Tirado B, Patel BV, Rein PJ	2014	Evaluation of an integrated adherence program aimed to increase Medicare Part D star rating measures	J Manag Care Spec Pharm. 2014;20(12):1193-203
Zullig LL, Melnyk SD, Stechuchak KM, McCant F, Danus S, Oddone E, Bastian L, Olsen M, Edelman D, Rakley S, Morey M, Bosworth HB	2014	The Cardiovascular Intervention Improvement Telemedicine Study (CITIES): rationale for a tailored behavioral and educational pharmacist-administered intervention for achieving cardiovascular disease risk reduction	Telemed J E Health. 2014;20(2):135-43
Fall E, Roche B, Izaute M, Batisse M, Tauveron I, Chakroun N.	2013	A brief psychological intervention to improve adherence in type 2 diabetes	Diabetes Metab. 2013;39(5):432-8
Insel KC, Einstein GO, Morrow DG, Hepworth JT	2013	A multifaceted prospective memory intervention to improve medication adherence: design of a randomized control trial	Contemp Clin Trials. 2013;34(1):45-52
Islam NS, Wyatt LC, Patel SD, Shapiro E, Tandon SD, Mukherji BR, Tanner M, Rey MJ, Trinh-Shevrin C	2013	Evaluation of a community health worker pilot intervention to improve diabetes management in Bangladeshi immigrants with type 2 diabetes in New York City	Diabetes Educ. 2013;39(4):478-93
Adhien P, van Dijk L, de Vegter M, Westein M, Nijpels G, Hugtenburg JG	2013	Evaluation of a pilot study to influence medication adherence of patients with diabetes mellitus type-2 by the pharmacy	Int J Clin Pharm. 2013;35(6):1113-9
Moskowitz D, Thom DH, Hessler D, Ghorob A, Bodenheimer T	2013	Peer coaching to improve diabetes self-management: which patients benefit most?	J Gen Intern Med. 2013;28(7):938-42
Mackenzie G, Ireland S, Moore S, Heinz I, Johnson R, Oczkowski W, Sahlas D	2013	Tailored interventions to improve hypertension management after stroke or TIA--phase II (TIMS II)	Can J Neurosci Nurs. 2013;35(1):27-34
Matsumoto PM, Barreto AR, Sakata KN, Siqueira YM, Zoboli EL, Fracolli LA	2012	[Health education in the care to clients of the blood glucose self-monitoring program	Rev Esc Enferm USP. 2012;46(3):761-5
Migneault JP, Dedier JJ, Wright JA, Heeren T, Campbell MK, Morisky DE, Rudd P, Friedman RH	2012	A culturally adapted telecommunication system to improve physical activity, diet quality, and medication adherence among hypertensive African-Americans: a randomized controlled trial	Ann Behav Med. 2012;43(1):62-73
Brennan TA, Dollear TJ, Hu M, Matlin OS, Shrank WH, Choudhry NK, Grambley W	2012	An integrated pharmacy-based program improved medication prescription and adherence rates in diabetes patients	Health Aff (Millwood). 2012;31(1):120-9
Gerber BS, Rapacki L, Castillo A, Tilton J, Touchette DR, Mihailescu D, Berbaum ML, Sharp LK	2012	Design of a trial to evaluate the impact of clinical pharmacists and community health promoters working with African-Americans and Latinos with diabetes	BMC Public Health. 2012;12:891
American Pharmacists Association	2012	DOTx. MED: Pharmacist-delivered interventions to improve care for patients with diabetes	J Am Pharm Assoc (2003). 2012;52(1):25-33
Ellis DA, Naar-King S, Chen X, Moltz K, Cunningham PB, Idalski-Carcone A	2012	Multisystemic therapy compared to telephone support for youth with poorly controlled diabetes: findings from a randomized controlled trial	Ann Behav Med. 2012;44(2:207-15
Zolfaghari M, Mousavifar SA, Pedram S, Haghani H	2012	The impact of nurse short message services and telephone follow-ups on diabetic adherence: which one is more effective?	J Clin Nurs. 2012;21(13-14):1922-31. Retraction in: J Clin Nurs. 2016;25(11-12):1781
Cooper LA, Roter DL, Carson KA, Bone LR, Larson SM, Miller ER 3rd, Barr MS, Levine DM	2011	A randomized trial to improve patient-centered care and hypertension control in underserved primary care patients	J Gen Intern Med. 2011;26(11):1297-304
Oberg EB, Bradley RD, Allen J, McCrory MA	2011	CAM: naturopathic dietary interventions for patients with type 2 diabetes	Complement Ther Clin Pract. 2011;17(3): 157-61
Jing S, Naliboff A, Kaufman MB, Choy M	2011	Descriptive analysis of mail interventions with physicians and patients to improve adherence with antihypertensive and antidiabetic medications in a mixed-model managed care organization of commercial and Medicare members	J Manag Care Pharm. 2011;17(5):355-66
Mitchell B, Armour C, Lee M, Song YJ, Stewart K, Peterson G, Hughes J, Smith L, Krass I	2011	Diabetes Medication Assistance Service: the pharmacist’s role in supporting patient self-management of type 2 diabetes (T2DM) in Australia	Patient Educ Couns. 2011;83(3):288-94
Labhardt ND, Balo JR, Ndam M, Manga E, Stoll B	2011	Improved retention rates with low-cost interventions in hypertension and diabetes management in a rural African environment of nurse-led care: a cluster-randomised trial	Trop Med Int Health. 2011;16(10):1276-84
Martin MY, Kim YI, Kratt P, Litaker MS, Kohler CL, Schoenberger YM, Clarke SJ, Prayor-Patterson H, Tseng TS, Pisu M, Williams OD	2011	Medication adherence among rural, low-income hypertensive adults: a randomized trial of a multimedia community-based intervention	Am J Health Promot. 2011;25(6):372-8
Morgado M, Rolo S, Castelo-Branco M	2011	Morgado M, Rolo S, Castelo-Branco M. Pharmacist intervention program to enhance hypertension control: a randomised controlled trial	Int J Clin Pharm. 2011;33(1):132-40
Griffin SJ, Simmons RK, Williams KM, Prevost AT, Hardeman W, Grant J, Whittle F, Boase S, Hobbis I, Brage S, Westgate K, Fanshawe T, Sutton S, Wareham NJ, Kinmonth AL; ADDITION-Plus study team.	2011	Protocol for the ADDITION-Plus study: a randomised controlled trial of an individually-tailored behaviour change intervention among people with recently diagnosed type 2 diabetes under intensive UK general practice care	BMC Public Health. 2011;11:211
Shah BR, Adams M, Peterson ED, Powers B, Oddone EZ, Royal K, McCant F, Grambow SC, Lindquist J, Bosworth HB	2011	Secondary prevention risk interventions via telemedicine and tailored patient education (SPRITE): a randomized trial to improve postmyocardial infarction management	Circ Cardiovasc Qual Outcomes. 2011;4(2):235-42
Carter BL, Doucette WR, Franciscus CL, Ardery G, Kluesner KM, Chrischilles EA	2010	Deterioration of blood pressure control after discontinuation of a physician-pharmacist collaborative intervention	Pharmacotherapy. 2010;30(3):228-35
Criswell TJ, Weber CA, Xu Y, Carter BL	2010	Effect of self-efficacy and social support on adherence to antihypertensive drugs	Pharmacotherapy. 2010;30(5):432-41
Lau R, Stewart K, McNamara KP, Jackson SL, Hughes JD, Peterson GM, Bortoletto DA, McDowell J, Bailey MJ, Hsueh A, George J	2010	Evaluation of a community pharmacy-based intervention for improving patient adherence to antihypertensives: a randomised controlled trial	BMC Health Serv Res. 2010;10:34
Robinson JD, Segal R, Lopez LM, Doty RE	2010	Impact of a pharmaceutical care intervention on blood pressure control in a chain pharmacy practice	Ann Pharmacother. 2010;44(1):88-96
Mishali M, Sominsky L, Heymann AD.	2010	Reducing resistance to diabetes treatment using short narrative interventions	Fam Pract. 2010;27(2):192-7
Lehmkuhl HD, Storch EA, Cammarata C, Meyer K, Rahman O, Silverstein J, Malasanos T, Geffken G	2010	Telehealth behavior therapy for the management of type 1 diabetes in adolescents	J Diabetes Sci Technol. 2010;4(1):199-208
Williams AF, Manias E, Walker RG	2010	The devil is in the detail - a multifactorial intervention to reduce blood pressure in co-existing diabetes and chronic kidney disease: a single blind, randomized controlled trial	BMC Fam Pract. 2010;11:3
Bonds DE, Hogan PE, Bertoni AG, Chen H, Clinch CR, Hiott AE, Rosenberger EL, Goff DC.	2009	A multifaceted intervention to improve blood pressure control: the Guideline Adherence for Heart Health (GLAD) study	Am Heart J. 2009;157(2):278-84
Feldman PH, McDonald MV, Mongoven JM, Peng TR, Gerber LM, Pezzin LE	2009	Home-based blood pressure interventions for blacks	Circ Cardiovasc Qual Outcomes. 2009;2(3):241-8
Dolor RJ, Yancy WS Jr, Owen WF, Matchar DB, Samsa GP, Pollak KI, Lin PH, Ard JD, Prempeh M, McGuire HL, Batch BC, Fan W, Svetkey LP	2009	Hypertension Improvement Project (HIP): study protocol and implementation challenges	Trials. 2009;10:13
Christie D, Strange V, Allen E, Oliver S, Wong IC, Smith F, Cairns J, Thompson R, Hindmarsh P, O’Neill S, Bull C, Viner R, Elbourne D	2009	Maximising engagement, motivation and long term change in a Structured Intensive Education Programme in Diabetes for children, young people and their families: Child and Adolescent Structured Competencies Approach to Diabetes Education (CASCADE)	BMC Pediatr. 2009;9:57
Bosworth HB, Olsen MK, Dudley T, Orr M, Goldstein MK, Datta SK, McCant F, Gentry P, Simel DL, Oddone EZ	2009	Patient education and provider decision support to control blood pressure in primary care: a cluster randomized trial	Am Heart J. 2009;157(3):450-6
Svarstad BL, Kotchen JM, Shireman TI, Crawford SY, Palmer PA, Vivian EM, Brown RL	2009	The Team Education and Adherence Monitoring (TEAM) trial: pharmacy interventions to improve hypertension control in blacks	Circ Cardiovasc Qual Outcomes. 2009;2(3):264-71
Green BB, Ralston JD, Fishman PA, Catz SL, Cook A, Carlson J, Tyll L, Carrell D, Thompson RS	2008	Electronic communications and home blood pressure monitoring (e-BP) study: design, delivery, and evaluation framework	Contemp Clin Trials. 2008;29(3):376-95
Farmer AJ, Prevost AT, Hardeman W, Craven A, Sutton S, Griffin SJ, Kinmonth AL; Support and Advice for Medication Trial Group	2008	Protocol for SAMS (Support and Advice for Medication Study): a randomised controlled trial of an intervention to support patients with type 2 diabetes with adherence to medication	BMC Fam Pract. 2008;9:20
Bosworth HB, Olsen MK, McCant F, Harrelson M, Gentry P, Rose C, Goldstein MK, Hoffman BB, Powers B, Oddone EZ	2007	Hypertension Intervention Nurse Telemedicine Study (HINTS): testing a multifactorial tailored behavioral/educational and a medication management intervention for blood pressure control	Am Heart J. 2007;153(6):918-24
Lin D, Hale S, Kirby E	2007	Improving diabetes management: structured clinic program for Canadian primary care	Can Fam Physician. 2007;53(1):73-7
Ellis DA, Naar-King S, Templin T, Frey MA, Cunningham PB	2007	Improving health outcomes among youth with poorly controlled type I diabetes: the role of treatment fidelity in a randomized clinical trial of multisystemic therapy	J Fam Psychol. 2007;21(3):363-71
Lin PH, Appel LJ, Funk K, Craddick S, Chen C, Elmer P, McBurnie MA, Champagne C	2007	The PREMIER intervention helps participants follow the Dietary Approaches to Stop Hypertension dietary pattern and the current Dietary Reference Intakes recommendations	J Am Diet Assoc. 2007;107(9):1541-51
Bosworth HB, Olsen MK, Dudley T, Orr M, Neary A, Harrelson M, Adams M, Svetkey LP, Dolor RJ, Oddone EZ	2007	The Take Control of Your Blood pressure (TCYB) study: study design and methodology	Contemp Clin Trials. 2007;28(1):33-47
Johnson SS, Driskell MM, Johnson JL, Prochaska JM, Zwick W, Prochaska JO	2006	Efficacy of a transtheoretical model-based expert system for antihypertensive adherence.	Dis Manag. 2006t;9(5):291-301
Roumie CL, Elasy TA, Greevy R, Griffin MR, Liu X, Stone WJ, Wallston KA, Dittus RS, Alvarez V, Cobb J, Speroff T	2006	Improving blood pressure control through provider education, provider alerts, and patient education: a cluster randomized trial	Ann Intern Med. 2006;145(3):165-75
Jenkins RG, Ornstein SM, Nietert PJ, Klockars SJ, Thiedke C	2006	Quality improvement for prevention of cardiovascular disease and stroke in an academicfamily medicine center: do racial differences in outcome exist?	Ethn Dis. 2006;16(1):132-7
Odegard PS, Goo A, Hummel J, Williams KL, Gray SL	2005	Caring for poorly controlled diabetes mellitus: a randomized pharmacist intervention	Ann Pharmacother. 2005;39(3):433-40
Szirmai LA, Arnold C, Farsang C	2005	Improving control of hypertension by an integrated approach -- results of the ‘Manage it well!’ programme	J Hypertens. 2005;23(1):203-11
Bosworth HB, Olsen MK, Goldstein MK, Orr M, Dudley T, McCant F, Gentry P, Oddone EZ	2005	The veterans’ study to improve the control of hypertension (V-STITCH): design and methodology	Contemp Clin Trials. 2005;26(2):155-68
Bailie RS, Si D, Robinson GW, Togni SJ, D’Abbs PH	2004	A multifaceted health-service intervention in remote Aboriginal communities: 3-year follow-up of the impact on diabetes care	Med J Aust. 2004;181(4):195-200
New JP, Mason JM, Freemantle N, Teasdale S, Wong L, Bruce NJ, Burns JA, Gibson JM	2004	Educational outreach in diabetes to encourage practice nurses to use primary care hypertension and hyperlipidaemia guidelines (EDEN): a randomized controlled trial	Diabet Med. 2004;21(6):599-603
Clark M, Hampson SE, Avery L, Simpson R	2004	Effects of a tailored lifestyle self-management intervention in patients with type 2 diabetes	Br J Health Psychol. 2004;9(Pt 3):365-79
Franklin V, Waller A, Pagliari C, Greene S	2003	“Sweet Talk”: text messaging support for intensive insulin therapy for young people with diabetes	Diabetes Technol Ther. 2003;5(6):991-6
Côté I, Grégoire JP, Moisan J, Chabot I, Lacroix G	2003	A pharmacy-based health promotion programme in hypertension: cost-benefit analysis	Pharmacoeconomics. 2003;21(6):415-28
Johnson BF, Hamilton G, Fink J, Lucey G, Bennet N, Lew R	2000	A design for testing interventions to improve adherence within a hypertension clinical trial	Control Clin Trials. 2000;21(1):62-72
Nyman MA, Murphy ME, Schryver PG, Naessens JM, Smith SA	2000	Improving performance in diabetes care: a multicomponent intervention	Eff Clin Pract. 2000;3(5):205-12

**Figure 1:**
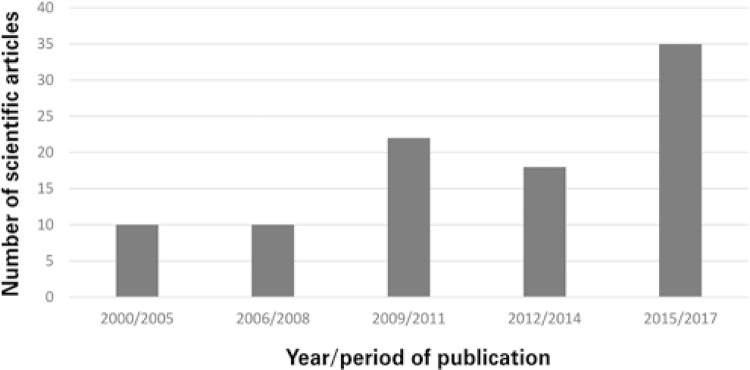
Number of articles listed on PubMed® according to period of publication

Data analysis revealed scientific production growth as of 2009, with a higher volume of publications describing interventions aimed to improve adherence to antihypertensive and antidiabetic therapy, between 2015 and 2017 ( Figure 1 ).

**Figure 2:**
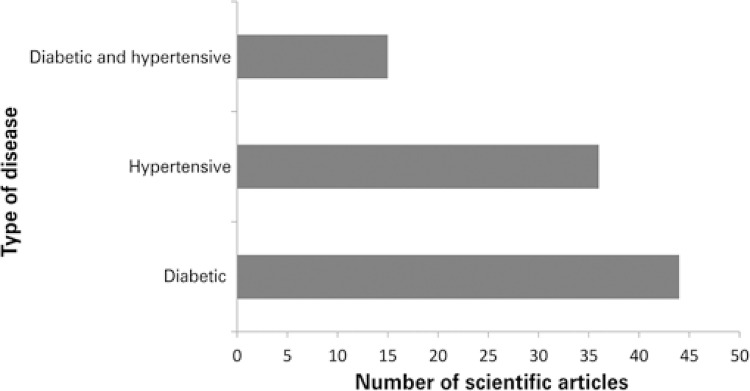
Number of scientific articles according to type of disease

Studies focusing on interventions aimed at diabetes patients were more abundant (46.3%; n=44), followed by articles describing interventions aimed at hypertension (37.9%; n=36). Only 15.8% (n=15) of articles described interventions aimed at both diseases ( Figure 2 ).

**Figure 3:**
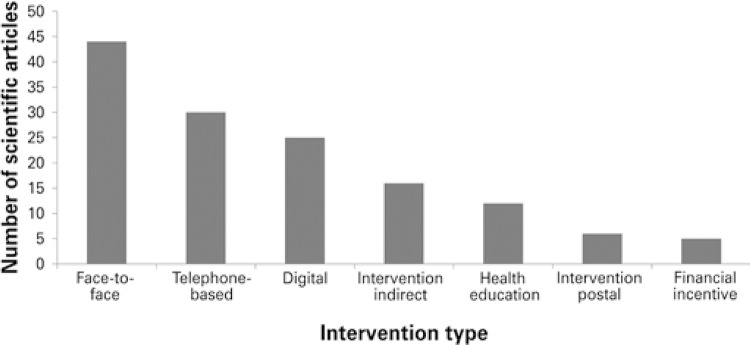
Thematic axis profile of scientific articles describing types of interventions aimed to improve adherence to antihypertensive and antidiabetic therapy

Most articles (63.2%; n=60) described a single intervention, whereas remaining articles included two (30.5%; n=29) or more (6.3%; n=6) concurrent interventions. Classification according to type of intervention was as follows: face-to-face, 46.3% (n=44); telephone-based, 31.6% (n=30); digital, 26.3% (n=25); indirect, 16.9% (n=16); health education, 12.7% (n=12); postal, 6.3% (n=6); and financial incentive, 5.2% (n=5) ( Figure 3 ).

Interventions were defined as follows: (1) face-to-face - individual appointments in clinics and home visits by health professionals; (2) telephone-based - whenever conducted over the telephone; (3) digital - SMS text messages, Apps or software *(* WhatsApp, etc.); (4) indirect - public policies, audits, guidelines and professional training; (5) health education - talks and orientations given to patients; (6) postal - letters sent by regular mail; and (7) financial incentive - payments made or discounts given for financial compensation of patients.

Article distribution according to continent of origin revealed larger numbers of studies conducted in North America (68.4%; n=65) or Europe (14.8%; n=14), with only a small proportion (3.1%; n=3) from South America.

## DISCUSSION

In this study, the findings derived from the analysis of scientific publications emphasize the importance of the topic selected in the realm of public policies aimed at health promotion, as shown by adoption of control measures by several counties in the face of increased prevalence of hypertension and diabetes.^9^ This process has led to the application of different interventions resulting in clinical improvement of patients and lower health care costs.^10^

The World Health Organization (WHO), driven by some countries, such as the United States, Canada, Australia and the United Kingdom, expected to reduce mortality rates associated with chronic diseases by 2% per year, up to 2015.^11^ With these estimates in mind, joint efforts by the WHO, governments, world organizations and the private sector approved the Global Action Plan for the Prevention and Control of NCDs 2013-2020. The WHO has also set voluntary targets for 2025, among which the reduction of premature mortality due to these diseases by 25%.^1^

The higher number of scientific publications on interventions in the last decade may reflect population aging, given physiological changes tend to increase with age, leading to higher prevalence of NCDs.^12^ Improved basic health care, growing urbanization, global commercialization of health harming products, and adoption of unhealthy life styles may have boosted scientific production during this period.^2^

Hypertension is a silent disease affecting individuals of all socioeconomic levels, with higher mortality and global prevalence rates compared to other NCDs;^5^ still, studies investigating interventions aimed at diabetic patients are even more abundant. This may be explained by the diversity of acute and chronic complications associated with *diabetes mellitus* and the two- to three-fold increase in health costs over the years as compared to costs associated with non-diabetic patients.^2^ In 2017, estimated global costs of diabetes amounted to US$ 850 billion, with significant social and economic impacts on healthcare systems.^2^

As regards different types of interventions promoting adherence to medical and non-medical antihypertensive and antidiabetic therapy, face-to-face interventions consisting of individual appointments and home visits were more commonly described in scientific literature.^13^ Individual appointments are widely used in outpatient services, clinics, community pharmacies, multidisciplinary health teams^14^ and other health centers, since they represent traditional methods involving joint analysis of barriers to adherence to therapy, and solutions for improved health outcomes, by physicians, pharmacists, nurses, psychologists, dietitians, physical educators and patients.^15^

Telephone-based interventions also proved to be efficient, since these encourage patients with several comorbidities to adopt best care practices via telephone call monitoring.^16^ This type of traditional intervention is widely used by pharmacists in community pharmacies and clinics; as drug managers, pharmacists provide guidance to patients regarding health behaviors, thereby contributing to improved adherence to medical and non-medical antihypertensive and antidiabetic therapy.^17^ Telephone-based services constitute more accessible alternatives, with reduced medical appointment load, lower transportation costs for low-income patients and the added benefit of proposing the insertion of personalized information.^18^

Digital interventions consisting of SMS text messages, Web, apps and WhatsApp have been attracting increasing attention in studies investigating adherence to treatment over the last few years. Such technology tools facilitate access to health information aimed to improve patients’ quality of life.^19^ One study has shown that combined technologies may encourage health behavior changes and increase adherence to antihypertensive and antidiabetic therapy.^20^

Digital interventions were shown to be particularly effective in underdeveloped and developing countries, for ensuring access to health benefits from remote areas worldwide via widely available technology.^21^ Mobile health tools, or m-Health, are a major trend in NCD control, given their low cost and ability to provide remote health care.^22^

Other interventions designed to improve health behavior in hypertensive and diabetic patients described in literature include indirect interventions, comprising public policies, health guidelines, audits and professional training.^23^

Health education interventions are often implemented by nurses and other health professionals by means of talks and group guidance sessions, particularly in communities where technological resources are limited or lacking.^24^ Studies describing postal interventions, consisting of letters containing health recommendations were scarce.^25^ Finally, financial incentive interventions applied by some health services, particularly the private sector, to encourage patient adherence out of financial compensation, were seldom described.^26^

As regards study origin, most scientific research related to interventions tailored to hypertensive and diabetic individuals were conducted in North America, followed by European countries. In the United States, for example, one in every three individuals, or 75.2 million American citizens suffer from hypertension, and almost half this population (35 million people) has blood pressure levels above recommendations.^27^ From 2011 to 2014, the US hypertension prevalence averaged 34% (34.5% and 33.4%, in men and women respectively); prevalence in the elderly population was 67.2%, with approximately 410,624 deaths due to primary or secondary causes, and a total cost of US$ 51.2 billion between 2012 and 2013.^28^

Diabetes affected approximately 30.3 million Americans in 2015, with 9.4% prevalence. Diabetes was the seventh cause of death in the country, with more than 252,806 deaths resulting directly or indirectly from the disease, annually.^29^ In 2017, total diabetes costs amounted to US$ 327 billion, with individuals aged 65 years or older accounting for most of the financial burden, and driving rising healthcare budget requirements.^2^ The 2010 estimated prevalence of diagnosed and undiagnosed diabetes in the adult population of 14% is expected increase to 21% up to 2050.^30^

Obesity is a major factor in the growing prevalence of other NCDs and has been associated with rising numbers of premature deaths due to hypertension and diabetes, particularly in countries such as the United States, where consumption of industrialized foods is high. Poor dietary habits resulted in 17% prevalence of obesity among children, and approximately one-third of the adult population (36.5%) affected between 2011 and 2014, particularly middle-aged individuals (40 to 59 years).^29^

The number of studies on interventions conducted in European countries has also increased. Population aging in these countries has led to a constant rise in NCD prevalence and sparked interest in strategies aimed at reducing the burden of health care costs.^31^

In South America, a continent comprising developing countries, studies investigating interventions aimed to increase adherence to antihypertensive and antidiabetic therapy are quite recent, and in lesser numbers compared to North America. In Brazil, improved health status and increased life expectancy translated into 18% growth of the aging population over the last 5 years, from 25.4 million in 2012, to 30 million in 2017. These factors contributed to growing estimated NCD prevalence over the course of one decade, with 14.2% increase in hypertension prevalence (from 22.5% to 25.7%, between 2006 and 2016) and 61.8% increase in diabetes prevalence (from 5.5% to 8.9%, between 2006 and 2016).^32^

High NCD prevalence in 2015 led to 424,058 deaths due to cardiovascular diseases, and 62,466 deaths due to diabetes,^33^ with total costs amounting to US$ 4.18 and US$ 22 billion, respectively.^6 , 9^ Over the last few years, lifestyle changes among Brazilian citizens have had significant impacts on obesity-related comorbidity rates, another important risk-factor for hypertension and diabetes.^5^ Obesity rates increased 60% in Brazil in 10 years, from 11.8% in 2006 to 18.9% in 2016, with higher prevalence (22.9%) among individuals aged 55 to 64 years.^32^

Brazil has adopted important measures to tackle NCDs over the last few years, such as establishing the *Sistema de Vigilância de Doenças Crônicas Não Transmissíveis* (VIGITEL) [Surveillance System for Non-communicable Chronic Diseases], for permanent monitoring of chronic diseases and assessment of the best intervention strategies.^32^ Creation of *Plano de Ações para o Enfrentamento de* DCNT 2011-2022 [Action Plan to Tackle NCDs 2011-2022] was another important measure encouraging the development of public policies aimed at health promotion, with significant contributions to the achievement of goals, such as 2% annual reduction in premature deaths due to NCDs until 2022,^6^ so as to ensure sustainable health development for the 2030 Agenda.^34^

Studies based on a single type of intervention were more common. In many countries, healthcare provision to elderly patients with two or more comorbidities poses a greater challenge to managers and health professionals. Interventions aimed to increase adherence to treatment require guidelines focused on incentives for patients in this age group.^35^ In the United States, delivery of multiple interventions to the same patient failed to improve health outcomes due to disease-specific requirements and higher costs. Therefore, comprehensive tools focused on patient quality of life rather than disease alone must be sought after.^35^

## CONCLUSION

This scientometric study revealed significant gaps. The number of studies conducted in Latin America, particularly in Brazil, was small, in contrast with the growing prevalence of hypertension and diabetes in the country and the Latin American continent overall. Financial incentive interventions were limited to some developed countries; still, despite limited evidence, this type of intervention may be a promising strategy for behavior change promotion. Studies investigating interventions designed to improve adherence to treatment by patients with comorbidities, such as hypertension and diabetes, were scarce.

Finally, traditional interventions such as face-to-face interviews or telephone calls were more commonly used to encourage adherence to antihypertensive and antidiabetic therapy, in spite of the current trend of digital technology application to leverage health behavior changes.
