# Supplementary material for: Scientometrics on interventions used for adherence of hypertension and diabetes therapies
Source: Einstein (Sao Paulo). 2019 Dec 6;18:eAO4723. doi: 10.31744/einstein_journal/2020AO4723 (PMC6905157; doi:10.31744/einstein_journal/2020AO4723)
Supplement: Translation

# Translation: Cienciometria em intervenções usadas para adesão ao tratamento de hipertensão e diabetes

**Authors:** Julio de Souza Sá, Lucas França Garcia, Marcelo Picinin Bernuci, Mirian Ueda Yamaguchi

**Affiliations:** 1 Centro Universitário de Maringá, Maringá, PR, Brasil.

**Keywords:** Promoção da saúde, Tratamento farmacológico, Hipertensão, Diabetes mellitus, Enfermagem em saúde comunitária, Assistência ambulatorial, Equipe interdisciplinar de saúde, Tecnologia biomédica, Bibliometria

## Abstract

**Objetivo:**

Identificar as intervenções utilizadas para melhorar a adesão de terapias medicamentosas e não medicamentosas de pacientes hipertensos e diabéticos.

**Métodos:**

Estudo cienciométrico realizado nos meses de fevereiro e março de 2018, utilizando os termos “intervenções para melhorar a adesão à terapia do diabetes”, “intervenções para melhorar a adesão à terapia de hipertensão” e “intervenções para melhorar a adesão à terapia da hipertensão e diabetes”, nas bases de dados PubMed^®^e SciELO.

**Resultados:**

Foram selecionados 95 artigos. A partir do ano 2009, observou-se crescimento da produção científica com maior volume entre os anos 2015 e 2017. O maior número de intervenções encontradas na literatura foi para pacientes com diabetes (46,31%). A intervenções mais utilizadas foram a face a face (46,31%), por chamada telefônica (31,58%) e a digital (26,31%). A América do Norte destacou-se no número de pesquisas, com 68,42% das publicações, seguida pela Europa, com 14,74%. Dentre os estudos, a maioria (63,16%) utilizou apenas um tipo de intervenção.

**Conclusão:**

Métodos de intervenção tradicionais foram mais empregados para promover a adesão às terapias anti-hipertensão e antidiabetes, embora o uso de tecnologias digitais desponte como tendência para melhorar esses comportamentos de saúde.

## INTRODUÇÃO

A hipertensão arterial e o *diabetes mellitus* estão entre as principais causas de mortes por doenças crônicas não transmissíveis (DCNT) em todo o mundo, com prevalência global de 22% para portadores de hipertensão, originando cerca de 9,4 milhões de mortes anualmente.^1^ Em indivíduos com diabetes, a prevalência mundial atinge aproximadamente 9% e tem provocado mais de 1,5 milhão de mortes todos os anos.^2^ Em vista disso, a adesão às terapias medicamentosa e não medicamentosa destas doenças desponta como importante desafio para a promoção da saúde.^3^

O crescimento das complicações por essas doenças é constante em países de média e baixa renda. Isso acontece, em parte, devido ao envelhecimento populacional e à necessidade de se adotar um estilo de vida saudável.^1^ A falta de adesão às terapias tem sinalizado um grande problema de saúde pública, no qual mais da metade dos pacientes não segue corretamente as terapias prescritas.^4^ A terapia medicamentosa para essas doenças é recomendada somente quando a terapia não medicamentosa, como dietas nutricionais, atividade física regular e outras práticas de estilo de vida saudável, não é suficiente.^5^

Diversas medidas têm sido adotadas para o enfrentamento das DCNT, definindo políticas e ações em todo o mundo.^6^ Os estudos de adesão às terapias apontam caminhos para o incentivo de estratégias inovadoras e intervenções comportamentais, que auxiliem no monitoramento adequado das terapias prescritas, proporcionando melhor qualidade de vida para os pacientes.^4^

As intervenções são definidas por ações de promoção da saúde capazes de promover mudança de comportamento na forma individual ou coletiva, considerando o contexto social em que o indivíduo está inserido, visando melhorar a adesão às terapias de DCNT.^7^ Existe uma variedade de intervenções que podem ajudar os gestores e os serviços de saúde no controle das DCNT, seja por meio de métodos tradicionais, como visitas domiciliares, aconselhamento individual ou em grupos, quanto pelo uso de tecnologias digitais, usadas para envio de lembretes ou conteúdos sobre comportamentos, no contexto da promoção da saúde.^8^

## OBJETIVO

Descrever o estado da arte das publicações científicas referentes ao desenvolvimento de intervenções utilizadas para melhorar a adesão às terapias anti-hipertensivas e antidiabéticas.

## MÉTODOS

Trata-se de um estudo cienciométrico, no qual a coleta dos dados foi realizada nos meses de fevereiro e março de 2018, nas bases de dados indexadas na PubMed^®^(https://www.ncbi.nlm.nih.gov/pubmed/) e *Scientific Electronic Library Online* (SciELO; https://www.scielo.org/).

Os termos utilizados para a pesquisa na base de dados PubMed^®^foram: “ *interventions to improve adherence to diabetes therapy* ”, “ *interventions to improve adherence to hypertension therapy* ” e “ *interventions to improve adherence to therapy for hypertension and diabetes* ”. Na base de dados SciELO foram utilizados os termos: “intervenções para melhorar a adesão à terapia do diabetes”, “intervenções para melhorar a adesão à terapia de hipertensão” e “intervenções para melhorar a adesão à terapia da hipertensão e diabetes”.

Foram excluídos os artigos de revisão de literatura e revisão sistemática de literatura, com intuito de alcançar maior precisão ao estudo, considerando apenas artigos originais. Após a análise dos títulos e resumos, os artigos incluídos foram classificados em sete temáticas sobre os tipos de intervenções: intervenção face a face; intervenção por chamada telefônica; intervenção digital; intervenção indireta; intervenção de educação em saúde; intervenção por Correios; e intervenção por incentivo financeiro.

Os dados foram organizados e tabulados em planilhas do programa Excel 2016, de acordo o ano das publicações, tipo de doença ou público-alvo, tipos de intervenções, quantidade de publicações por continente e número de intervenções por estudo.

## RESULTADOS

Foram encontradas 600 publicações entre os anos de 2000 a 2018 com os descritores do estudo no PubMed^®^, e nenhum artigo foi encontrado na base de SciELO. Após a leitura dos títulos e resumos para verificar a adequação aos objetivos do estudo, 95 artigos foram selecionados para análise ( Tabela 1 - Apêndice 1). Foram excluídos 505 artigos por não atenderem os critérios de inclusão do estudo.

**Tabela 1:** Artigos incluídos no estudo

Autores	Ano	Título do artigo	Periódico
Monroe AK, Pena JS, Moore RD, Riekert KA, Eakin MN, Kripalani S, Chander G	2018	*Randomized controlled trial of a pictorial aid intervention for medication adherence among HIV-positive patients with comorbid diabetes or hypertension*	AIDS Care. 2018;30(2):199-206
do Valle Nascimento TM, Resnicow K, Nery M, Brentani A, Kaselitz E, Agrawal P, Mand S, Heisler M	2017	*A pilot study of a Community Health Agent-led type 2 diabetes self-management program using Motivational Interviewing-based approaches in a public primary care center in São Paulo, Brazil*	BMC Health Serv Res. 2017;17(1):32
Wong CA, Miller VA, Murphy K, Small D, Ford CA, Willi SM, Feingold J, Morris A, Ha YP, Zhu J, Wang W, Patel MS	2017	*Effect of Financial Incentives on Glucose Monitoring Adherence and Glycemic Control Among Adolescents and Young Adults With Type 1 Diabetes: A Randomized Clinical Trial*	JAMA Pediatr. 2017;171(12):1176-83
Frias J, Virdi N, Raja P, Kim Y, Savage G, Osterberg L	2017	*Effectiveness of Digital Medicines to Improve Clinical Outcomes in Patients with Uncontrolled Hypertension and Type 2 Diabetes: Prospective, Open-Label, Cluster-Randomized Pilot Clinical Trial*	J Med Internet Res. 2017;19(7):e246
Davis SA, Carpenter D, Cummings DM, Lee C, Blalock SJ, Scott JE, Rodebaugh L, Ferreri SP, Sleath B	2017	*Patient adoption of an internet based diabetes medication tool to improve adherence: a pilot study*	Patient Educ Couns. 2017;100(1):174-8
Floyd BD, Block JM, Buckingham BB, Ly T, Foster N, Wright R, Mueller CL, Hood KK, Shah AC	2017	*Stabilization of glycemic control and improved quality of life using a shared medical appointment model in adolescents with type 1 diabetes in suboptimal control*	Pediatr Diabetes. 2017;18(3):204-12
Lewey J, Wei W, Lauffenburger JC, Makanji S, Chant A, DiGeronimo J, Nanchanatt G, Jan S, Choudhry NK	2017	*Targeted Adherence Intervention to Reach Glycemic Control with Insulin Therapy for patients with Diabetes (TARGIT-Diabetes): rationale and design of a pragmatic randomised clinical trial*	BMJ Open. 2017;7(10):e016551
Di Bartolo P, Nicolucci A, Cherubini V, Iafusco D, Scardapane M, Rossi MC	2017	*Young patients with type 1 diabetes poorly controlled and poorly compliant with self-monitoring of blood glucose: can technology help? Results of the i-NewTrend randomized clinical trial*	Acta Diabetol. 2017;54(4):393-402
Albini F, Xiaoqiu Liu, Torlasco C, Soranna D, Faini A, Ciminaghi R, Celsi A, Benedetti M, Zambon A, di Rienzo M, Parati G	2016	*An ICT and mobile health integrated approach to optimize patients’ education on hypertension and its management by physicians: The Patients Optimal Strategy of Treatment(POST) pilot study*	Conf Proc IEEE Eng Med Biol Soc. 2016;2016:517-20
Nelson LA, Mulvaney SA, Gebretsadik T, Ho YX, Johnson KB, Osborn CY	2016	*Disparities in the use of a mHealth medication adherence promotion intervention for low-income adults with type 2 diabetes*	J Am Med Inform Assoc. 2016;23(1):12-8
Vissenberg C, Stronks K, Nijpels G, Uitewaal PJ, Middelkoop BJ, Kohinor MJ, Hartman MA, Nierkens V	2016	*Impact of a social network-based intervention promoting diabetes self-management in socioeconomically deprived patients: a qualitative evaluation of the intervention strategies*	BMJ Open. 2016;6(4):e010254
Choudhry NK, Isaac T, Lauffenburger JC, Gopalakrishnan C, Khan NF, Lee M, Vachon A, Iliadis TL, Hollands W, Doheny S, Elman S, Kraft JM, Naseem S, Gagne JJ, Jackevicius CA, Fischer MA, Solomon DH, Sequist TD	2016	*Rationale and design of the Study of a Tele-pharmacy Intervention for Chronic diseases to Improve Treatment adherence (STIC2IT): A cluster-randomized pragmatic trial*	Am Heart J. 2016;180:90-7
Piette JD, Marinec N, Janda K, Morgan E, Schantz K, Yujra AC, Pinto B, Soto JM, Janevic M, Aikens JE	2016	*Structured Caregiver Feedback Enhances Engagement and Impact of Mobile Health Support: A Randomized Trial in a Lower-Middle-Income Country*	Telemed J E Health. 2016;22(4):261-8
Lynch CP, Williams JS, J Ruggiero K, G Knapp R, Egede LE	2016	*Tablet-Aided BehavioraL intervention EffecT on Self-management skills (TABLETS) for Diabetes*	Trials. 2016;17:157
Kravetz JD, Walsh RF	2016	*Team-Based Hypertension Management to Improve Blood Pressure Control*	J Prim Care Community Health. 2016;7(4):272-5
Mayberry LS, Berg CA, Harper KJ, Osborn CY	2016	*The Design, Usability, and Feasibility of a Family-Focused Diabetes Self-Care Support mHealth Intervention for Diverse, Low-Income Adults with Type 2 Diabetes*	J Diabetes Res. 2016;2016:7586385
Lin TY, Chen CY, Huang YT, Ting MK, Huang JC, Hsu KH	2016	*The effectiveness of a pay for performance program on diabetes care in Taiwan: a nationwide population-based longitudinal study*	Health Policy. 2016;120(11):1313-21
Reese PP, Kessler JB, Doshi JA, Friedman J, Mussell AS, Carney C, Zhu J, Wang W, Troxel A, Young P, Lawnicki V, Rajpathak S, Volpp K	2016	*Two Randomized Controlled Pilot Trials of Social Forces to Improve Statin Adherence among Patients with Diabetes*	J Gen Intern Med. 2016;31(4):402-10
Duke DC, Wagner DV, Ulrich J, Freeman KA, Harris MA	2016	*Videoconferencing for Teens With Diabetes: Family Matters*	J Diabetes Sci Technol. 2016;10(4):816-23
Schoenthaler A, De La Calle F, Barrios-Barrios M, Garcia A, Pitaro M, Lum A, Rosal M	2015	*A practice-based randomized controlled trial to improve medication adherence among Latinos with hypertension: study protocol for a randomized controlled trial*	Trials. 2015;16:290
Volpp KG, Troxel AB, Long JA, Ibrahim SA, Appleby D, Smith JO, Jaskowiak J, Helweg-Larsen M, Doshi JA, Kimmel SE	2015	*A randomized controlled trial of co-payment elimination: the CHORD trial.* [ClinicalTrials.gov NCT00133068].	Am J Manag Care. 2015;21(8):e455-64
Volpp KG, Troxel AB, Long JA, Ibrahim SA, Appleby D, Smith JO, Jaskowiak J, Helweg-Larsen M, Doshi JA, Kimmel SE	2015	*A randomized controlled trial of negative co-payments: the CHORD trial*	Am J Manag Care. 2015;21(8):e465-73
Fischer MA, Jones JB, Wright E, Van Loan RP, Xie J, Gallagher L, Wurst AM, Shrank WH	2015	A randomized telephone intervention trial to reduce primary medication nonadherence	J Manag Care Spec Pharm. 2015;21(2):124-31
Margolis KL, Asche SE, Bergdall AR, Dehmer SP, Maciosek MV, Nyboer RA, O’Connor PJ, Pawloski PA, Sperl-Hillen JM, Trower NK, Tucker AD, Green BB	2015	*A Successful Multifaceted Trial to Improve Hypertension Control in Primary Care: Why Did it Work?*	J Gen Intern Med. 2015;30(11):1665-72
Weiss DM, Casten RJ, Leiby BE, Hark LA, Murchison AP, Johnson D, Stratford S, Henderer J, Rovner BW, Haller JA	2015	*Effect of Behavioral Intervention on Dilated Fundus Examination Rates in Older African American Individuals With Diabetes Mellitus: a Randomized Clinical Trial*	JAMA Ophthalmol. 2015;133(9):1005-12
Xin C, Xia Z, Jiang C, Lin M, Li G	2015	*Effect of pharmaceutical care on medication adherence of patients newly prescribed insulin therapy: a randomized controlled study*	Patient Prefer Adherence. 2015;9:797-802
Fortuna RJ, Nagel AK, Rose E, McCann R, Teeters JC, Quigley DD, Bisognano JD, Legette-Sobers S, Liu C, Rocco TA	2015	*Effectiveness of a multidisciplinary intervention to improve hypertension control in an urban underserved practice*	J Am Soc Hypertens. 2015;9(12):966-74
Friedberg JP, Rodriguez MA, Watsula ME, Lin I, Wylie-Rosett J, Allegrante JP, Lipsitz SR, Natarajan S	2015	*Effectiveness of a tailored behavioral intervention to improve hypertension control: primary outcomes of a randomized controlled trial*	Hypertension. 2015;65(2):440-6
Wayne N, Perez DF, Kaplan DM, Ritvo P	2015	*Health Coaching Reduces HbA1c in Type 2 Diabetic Patients From a Lower-Socioeconomic Status Community: A Randomized Controlled Trial*	J Med Internet Res. 2015;17(10):e224
Leon N, Surender R, Bobrow K, Muller J, Farmer A	2015	*Improving treatment adherence for blood pressure lowering via mobile phone SMS-messages in South Africa: a qualitative evaluation of the SMS-text Adherence SuppoRt (StAR) trial*	BMC Fam Pract. 2015;16:80
Johnson RM, Johnson T, Zimmerman SD, Marsh GM, Garcia-Dominic O	2015	*Outcomes of a Seven Practice Pilot in a Pay-For-Performance (P4P)-Based Program in Pennsylvania*	J Racial Ethn Health Disparities. 2015;2(1):139-48
Shane-McWhorter L, McAdam-Marx C, Lenert L, Petersen M, Woolsey S, Coursey JM, Whittaker TC, Hyer C, LaMarche D, Carroll P, Chuy L	2015	*Pharmacist-provided diabetes management and education via a telemonitoring program*	J Am Pharm Assoc (2003). 2015;55(5):516-26
Kjeldsen LJ, Bjerrum L, Dam P, Larsen BO, Rossing C, Søndergaard B, Herborg H	2015	*Safe and effective use of medicines for patients with type 2 diabetes – A randomized controlled trial of two interventions delivered by local pharmacies*	Res Social Adm Pharm. 2015;11(1):47-62
Chamany S, Walker EA, Schechter CB, Gonzalez JS, Davis NJ, Ortega FM, Carrasco J, Basch CE, Silver LD	2015	*Telephone Intervention to Improve Diabetes Control: A Randomized Trial in the New York City A1c Registry*	Am J Prev Med. 2015;49(6):832-41
Cassimatis M, Kavanagh DJ, Hills AP, Smith AC, Scuffham PA, Gericke C, Parham S	2015	*The OnTrack Diabetes Web-Based Program for Type 2 Diabetes and Dysphoria Self-Management: a Randomized Controlled Trial Protocol*	JMIR Res Protoc. 2015;4(3):e97
Baynouna LM, Neglekerke NJ, Ali HE, ZeinAlDeen SM, Al Ameri TA	2014	*Audit of healthy lifestyle behaviors among patients with diabetes and hypertension attending ambulatory health care services in the United Arab Emirates*	Glob Health Promot. 2014;21(4):44-51
Jaser SS, Patel N, Linsky R, Whittemore R	2014	*Development of a positive psychology intervention to improve adherence in adolescents with type 1 diabetes*	J Pediatr Health Care. 2014;28(6):478-85
Bobrow K, Brennan T, Springer D, Levitt NS, Rayner B, Namane M, Yu LM, Tarassenko L, Farmer A	2014	*Efficacy of a text messaging (SMS) based intervention for adults with hypertension: protocol for the StAR (SMS Text-message Adherence suppoRt trial) randomised controlled trial*	BMC Public Health. 2014;14:28
Leslie RS, Tirado B, Patel BV, Rein PJ	2014	Evaluation of an integrated adherence program aimed to increase Medicare Part D star rating measures	J Manag Care Spec Pharm. 2014;20(12):1193-203
Zullig LL, Melnyk SD, Stechuchak KM, McCant F, Danus S, Oddone E, Bastian L, Olsen M, Edelman D, Rakley S, Morey M, Bosworth HB	2014	*The Cardiovascular Intervention Improvement Telemedicine Study (CITIES): rationale for a tailored behavioral and educational pharmacist-administered intervention for achieving cardiovascular disease risk reduction*	Telemed J E Health. 2014;20(2):135-43
Fall E, Roche B, Izaute M, Batisse M, Tauveron I, Chakroun N.	2013	*A brief psychological intervention to improve adherence in type 2 diabetes*	Diabetes Metab. 2013;39(5):432-8
Insel KC, Einstein GO, Morrow DG, Hepworth JT	2013	*A multifaceted prospective memory intervention to improve medication adherence: design of a randomized control trial*	Contemp Clin Trials. 2013;34(1):45-52
Islam NS, Wyatt LC, Patel SD, Shapiro E, Tandon SD, Mukherji BR, Tanner M, Rey MJ, Trinh-Shevrin C	2013	Evaluation of a community health worker pilot intervention to improve diabetes management in Bangladeshi immigrants with type 2 diabetes in New York City	Diabetes Educ. 2013;39(4):478-93
Adhien P, van Dijk L, de Vegter M, Westein M, Nijpels G, Hugtenburg JG	2013	*Evaluation of a pilot study to influence medication adherence of patients with diabetes mellitus type-2 by the pharmacy*	Int J Clin Pharm. 2013;35(6):1113-9
Moskowitz D, Thom DH, Hessler D, Ghorob A, Bodenheimer T	2013	*Peer coaching to improve diabetes self-management: which patients benefit most?*	J Gen Intern Med. 2013;28(7):938-42
Mackenzie G, Ireland S, Moore S, Heinz I, Johnson R, Oczkowski W, Sahlas D	2013	*Tailored interventions to improve hypertension management after stroke or TIA--phase II (TIMS II)*	Can J Neurosci Nurs. 2013;35(1):27-34
Matsumoto PM, Barreto AR, Sakata KN, Siqueira YM, Zoboli EL, Fracolli LA	2012	*[Health education in the care to clients of the blood glucose self-monitoring program*	Rev Esc Enferm USP. 2012;46(3):761-5
Migneault JP, Dedier JJ, Wright JA, Heeren T, Campbell MK, Morisky DE, Rudd P, Friedman RH	2012	*A culturally adapted telecommunication system to improve physical activity, diet quality, and medication adherence among hypertensive African-Americans: a randomized controlled trial*	Ann Behav Med. 2012;43(1):62-73
Brennan TA, Dollear TJ, Hu M, Matlin OS, Shrank WH, Choudhry NK, Grambley W	2012	*An integrated pharmacy-based program improved medication prescription and adherence rates in diabetes patients*	Health Aff (Millwood). 2012;31(1):120-9
Gerber BS, Rapacki L, Castillo A, Tilton J, Touchette DR, Mihailescu D, Berbaum ML, Sharp LK	2012	*Design of a trial to evaluate the impact of clinical pharmacists and community health promoters working with African-Americans and Latinos with diabetes*	BMC Public Health. 2012;12:891
American Pharmacists Association	2012	*DOTx. MED: Pharmacist-delivered interventions to improve care for patients with diabetes*	J Am Pharm Assoc (2003). 2012;52(1):25-33
Ellis DA, Naar-King S, Chen X, Moltz K, Cunningham PB, Idalski-Carcone A	2012	Multisystemic therapy compared to telephone support for youth with poorly controlled diabetes: findings from a randomized controlled trial	Ann Behav Med. 2012;44(2:207-15
Zolfaghari M, Mousavifar SA, Pedram S, Haghani H	2012	*The impact of nurse short message services and telephone follow-ups on diabetic adherence: which one is more effective?*	J Clin Nurs. 2012;21(13-14):1922-31. Retraction in: J Clin Nurs. 2016;25(11-12):1781
Cooper LA, Roter DL, Carson KA, Bone LR, Larson SM, Miller ER 3rd, Barr MS, Levine DM	2011	*A randomized trial to improve patient-centered care and hypertension control in underserved primary care patients*	J Gen Intern Med. 2011;26(11):1297-304
Oberg EB, Bradley RD, Allen J, McCrory MA	2011	*CAM: naturopathic dietary interventions for patients with type 2 diabetes*	Complement Ther Clin Pract. 2011;17(3): 157-61
Jing S, Naliboff A, Kaufman MB, Choy M	2011	*Descriptive analysis of mail interventions with physicians and patients to improve adherence with antihypertensive and antidiabetic medications in a mixed-model managed care organization of commercial and Medicare members*	J Manag Care Pharm. 2011;17(5):355-66
Mitchell B, Armour C, Lee M, Song YJ, Stewart K, Peterson G, Hughes J, Smith L, Krass I	2011	*Diabetes Medication Assistance Service: the pharmacist’s role in supporting patient self-management of type 2 diabetes (T2DM) in Australia*	Patient Educ Couns. 2011;83(3):288-94
Labhardt ND, Balo JR, Ndam M, Manga E, Stoll B	2011	*Improved retention rates with low-cost interventions in hypertension and diabetes management in a rural African environment of nurse-led care: a cluster-randomised trial*	Trop Med Int Health. 2011;16(10):1276-84
Martin MY, Kim YI, Kratt P, Litaker MS, Kohler CL, Schoenberger YM, Clarke SJ, Prayor-Patterson H, Tseng TS, Pisu M, Williams OD	2011	*Medication adherence among rural, low-income hypertensive adults: a randomized trial of a multimedia community-based intervention*	Am J Health Promot. 2011;25(6):372-8
Morgado M, Rolo S, Castelo-Branco M	2011	*Morgado M, Rolo S, Castelo-Branco M. Pharmacist intervention program to enhance hypertension control: a randomised controlled trial*	Int J Clin Pharm. 2011;33(1):132-40
Griffin SJ, Simmons RK, Williams KM, Prevost AT, Hardeman W, Grant J, Whittle F, Boase S, Hobbis I, Brage S, Westgate K, Fanshawe T, Sutton S, Wareham NJ, Kinmonth AL; ADDITION-Plus study team.	2011	*Protocol for the ADDITION-Plus study: a randomised controlled trial of an individually-tailored behaviour change intervention among people with recently diagnosed type 2 diabetes under intensive UK general practice care*	BMC Public Health. 2011;11:211
Shah BR, Adams M, Peterson ED, Powers B, Oddone EZ, Royal K, McCant F, Grambow SC, Lindquist J, Bosworth HB	2011	*Secondary prevention risk interventions via telemedicine and tailored patient education (SPRITE): a randomized trial to improve postmyocardial infarction management*	Circ Cardiovasc Qual Outcomes. 2011;4(2):235-42
Carter BL, Doucette WR, Franciscus CL, Ardery G, Kluesner KM, Chrischilles EA	2010	*Deterioration of blood pressure control after discontinuation of a physician-pharmacist collaborative intervention*	Pharmacotherapy. 2010;30(3):228-35
Criswell TJ, Weber CA, Xu Y, Carter BL	2010	*Effect of self-efficacy and social support on adherence to antihypertensive drugs*	Pharmacotherapy. 2010;30(5):432-41
Lau R, Stewart K, McNamara KP, Jackson SL, Hughes JD, Peterson GM, Bortoletto DA, McDowell J, Bailey MJ, Hsueh A, George J	2010	*Evaluation of a community pharmacy-based intervention for improving patient adherence to antihypertensives: a randomised controlled trial*	BMC Health Serv Res. 2010;10:34
Robinson JD, Segal R, Lopez LM, Doty RE	2010	*Impact of a pharmaceutical care intervention on blood pressure control in a chain pharmacy practice*	Ann Pharmacother. 2010;44(1):88-96
Mishali M, Sominsky L, Heymann AD.	2010	*Reducing resistance to diabetes treatment using short narrative interventions*	Fam Pract. 2010;27(2):192-7
Lehmkuhl HD, Storch EA, Cammarata C, Meyer K, Rahman O, Silverstein J, Malasanos T, Geffken G	2010	*Telehealth behavior therapy for the management of type 1 diabetes in adolescents*	J Diabetes Sci Technol. 2010;4(1):199-208
Williams AF, Manias E, Walker RG	2010	*The devil is in the detail - a multifactorial intervention to reduce blood pressure in co-existing diabetes and chronic kidney disease: a single blind, randomized controlled trial*	BMC Fam Pract. 2010;11:3
Bonds DE, Hogan PE, Bertoni AG, Chen H, Clinch CR, Hiott AE, Rosenberger EL, Goff DC.	2009	*A multifaceted intervention to improve blood pressure control: the Guideline Adherence for Heart Health (GLAD) study*	Am Heart J. 2009;157(2):278-84
Feldman PH, McDonald MV, Mongoven JM, Peng TR, Gerber LM, Pezzin LE	2009	*Home-based blood pressure interventions for blacks*	Circ Cardiovasc Qual Outcomes. 2009;2(3):241-8
Dolor RJ, Yancy WS Jr, Owen WF, Matchar DB, Samsa GP, Pollak KI, Lin PH, Ard JD, Prempeh M, McGuire HL, Batch BC, Fan W, Svetkey LP	2009	*Hypertension Improvement Project (HIP): study protocol and implementation challenges*	Trials. 2009;10:13
Christie D, Strange V, Allen E, Oliver S, Wong IC, Smith F, Cairns J, Thompson R, Hindmarsh P, O’Neill S, Bull C, Viner R, Elbourne D	2009	Maximising engagement, motivation and long term change in a Structured Intensive Education Programme in Diabetes for children, young people and their families: Child and Adolescent Structured Competencies Approach to Diabetes Education (CASCADE)	BMC Pediatr. 2009;9:57
Bosworth HB, Olsen MK, Dudley T, Orr M, Goldstein MK, Datta SK, McCant F, Gentry P, Simel DL, Oddone EZ	2009	*Patient education and provider decision support to control blood pressure in primary care: a cluster randomized trial*	Am Heart J. 2009;157(3):450-6
Svarstad BL, Kotchen JM, Shireman TI, Crawford SY, Palmer PA, Vivian EM, Brown RL	2009	*The Team Education and Adherence Monitoring (TEAM) trial: pharmacy interventions to improve hypertension control in blacks*	Circ Cardiovasc Qual Outcomes. 2009;2(3):264-71
Green BB, Ralston JD, Fishman PA, Catz SL, Cook A, Carlson J, Tyll L, Carrell D, Thompson RS	2008	*Electronic communications and home blood pressure monitoring (e-BP) study: design, delivery, and evaluation framework*	Contemp Clin Trials. 2008;29(3):376-95
Farmer AJ, Prevost AT, Hardeman W, Craven A, Sutton S, Griffin SJ, Kinmonth AL; Support and Advice for Medication Trial Group	2008	*Protocol for SAMS (Support and Advice for Medication Study): a randomised controlled trial of an intervention to support patients with type 2 diabetes with adherence to medication*	BMC Fam Pract. 2008;9:20
Bosworth HB, Olsen MK, McCant F, Harrelson M, Gentry P, Rose C, Goldstein MK, Hoffman BB, Powers B, Oddone EZ	2007	*Hypertension Intervention Nurse Telemedicine Study (HINTS): testing a multifactorial tailored behavioral/educational and a medication management intervention for blood pressure control*	Am Heart J. 2007;153(6):918-24
Lin D, Hale S, Kirby E	2007	*Improving diabetes management: structured clinic program for Canadian primary care*	Can Fam Physician. 2007;53(1):73-7
Ellis DA, Naar-King S, Templin T, Frey MA, Cunningham PB	2007	Improving health outcomes among youth with poorly controlled type I diabetes: the role of treatment fidelity in a randomized clinical trial of multisystemic therapy	J Fam Psychol. 2007;21(3):363-71
Lin PH, Appel LJ, Funk K, Craddick S, Chen C, Elmer P, McBurnie MA, Champagne C	2007	*The PREMIER intervention helps participants follow the Dietary Approaches to Stop Hypertension dietary pattern and the current Dietary Reference Intakes recommendations*	J Am Diet Assoc. 2007;107(9):1541-51
Bosworth HB, Olsen MK, Dudley T, Orr M, Neary A, Harrelson M, Adams M, Svetkey LP, Dolor RJ, Oddone EZ	2007	*The Take Control of Your Blood pressure (TCYB) study: study design and methodology*	Contemp Clin Trials. 2007;28(1):33-47
Johnson SS, Driskell MM, Johnson JL, Prochaska JM, Zwick W, Prochaska JO	2006	*Efficacy of a transtheoretical model-based expert system for antihypertensive adherence.*	Dis Manag. 2006t;9(5):291-301
Roumie CL, Elasy TA, Greevy R, Griffin MR, Liu X, Stone WJ, Wallston KA, Dittus RS, Alvarez V, Cobb J, Speroff T	2006	*Improving blood pressure control through provider education, provider alerts, and patient education: a cluster randomized trial*	Ann Intern Med. 2006;145(3):165-75
Jenkins RG, Ornstein SM, Nietert PJ, Klockars SJ, Thiedke C	2006	*Quality improvement for prevention of cardiovascular disease and stroke in an academicfamily medicine center: do racial differences in outcome exist?*	Ethn Dis. 2006;16(1):132-7
Odegard PS, Goo A, Hummel J, Williams KL, Gray SL	2005	*Caring for poorly controlled diabetes mellitus: a randomized pharmacist intervention*	Ann Pharmacother. 2005;39(3):433-40
Szirmai LA, Arnold C, Farsang C	2005	*Improving control of hypertension by an integrated approach -- results of the ‘Manage it well!’ programme*	J Hypertens. 2005;23(1):203-11
Bosworth HB, Olsen MK, Goldstein MK, Orr M, Dudley T, McCant F, Gentry P, Oddone EZ	2005	*The veterans’ study to improve the control of hypertension (V-STITCH): design and methodology*	Contemp Clin Trials. 2005;26(2):155-68
Bailie RS, Si D, Robinson GW, Togni SJ, D’Abbs PH	2004	*A multifaceted health-service intervention in remote Aboriginal communities: 3-year follow-up of the impact on diabetes care*	Med J Aust. 2004;181(4):195-200
New JP, Mason JM, Freemantle N, Teasdale S, Wong L, Bruce NJ, Burns JA, Gibson JM	2004	*Educational outreach in diabetes to encourage practice nurses to use primary care hypertension and hyperlipidaemia guidelines (EDEN): a randomized controlled trial*	Diabet Med. 2004;21(6):599-603
Clark M, Hampson SE, Avery L, Simpson R	2004	*Effects of a tailored lifestyle self-management intervention in patients with type 2 diabetes*	Br J Health Psychol. 2004;9(Pt 3):365-79
Franklin V, Waller A, Pagliari C, Greene S	2003	*“Sweet Talk”: text messaging support for intensive insulin therapy for young people with diabetes*	Diabetes Technol Ther. 2003;5(6):991-6
Côté I, Grégoire JP, Moisan J, Chabot I, Lacroix G	2003	*A pharmacy-based health promotion programme in hypertension: cost-benefit analysis*	Pharmacoeconomics. 2003;21(6):415-28
Johnson BF, Hamilton G, Fink J, Lucey G, Bennet N, Lew R	2000	*A design for testing interventions to improve adherence within a hypertension clinical trial*	Control Clin Trials. 2000;21(1):62-72
Nyman MA, Murphy ME, Schryver PG, Naessens JM, Smith SA	2000	*Improving performance in diabetes care: a multicomponent intervention*	Eff Clin Pract. 2000;3(5):205-12

Identificou-se um crescimento na produção científica a partir do ano 2009, obtendo maior volume entre os anos 2015/2017, período em que houve maior número de publicações referentes às intervenções para melhorar a adesão à terapia da hipertensão e do diabetes ( Figura 1 ).

**Figura 1:** Número de artigos publicados nas bases de dados PubMed® conforme o período de publicação

Ainda, foi encontrado número maior de estudos associados a intervenções para pacientes com diabetes, correspondendo a 46,3% (n=44), seguido de 37,9% (n=36) relacionados à intervenções para hipertensão. Apenas 15,8% (n=15) das publicações eram de intervenções para as duas doenças ( Figura 2 ).

**Figura 2:** Número de produções científicas conforme o tipo de doença

Com relação ao número de intervenções estudadas, 63,2% (n=60) continham apenas um tipo de intervenção, 30,5% (n=29) duas intervenções, e somente 6,3% (n=6) incluíram mais de duas intervenções concomitantes. Com relação ao tipo de intervenção, 46,3% (n=44) dos estudos apresentaram intervenção face a face, 31,6% (n=30) intervenção por chamada telefônica, 26,3% (n=25) intervenção digital, 16,9% (n=16) intervenção indireta, 12,7% (n=12) intervenção de educação em saúde, 6,3% (n=6) intervenção por Correios e 5,2% (n=5) intervenção com incentivo financeiro ( Figura 3 ).

**Figura 3:** Perfil dos eixos temáticos da produção científica relacionada os tipos de intervenções para melhorar a adesão à terapia da hipertensão e do diabetes

As intervenções mencionadas puderam ser caracterizadas da seguinte maneira: (1) face a face, se consulta individual em clínicas e visitas domiciliares realizadas por profissionais da saúde; (2) chamada telefônica, se ligações via telefone; (3) digital, se envio de mensagens de texto SMS e aplicativos (App ou software), tais como WhatsApp; (4) indireta, se políticas públicas, auditorias, diretrizes e treinamento de profissionais envolvidos; (5) educação em saúde, se palestras e orientações aos pacientes; (6) Correios, se envio de cartas via Correios; e (7) incentivo financeiro, se realização pagamentos ou descontos promovendo compensação financeira aos pacientes.

A distribuição dos artigos de acordo com o continente onde o estudo foi realizado indicou que grande parte da produção era da América do Norte, 68,4% (n=65), seguida por Europa, com 14,8% (n=14); e apenas 3,1% (n=3) na América do Sul.

## DISCUSSÃO

A apreciação dos estudos encontrados na literatura científica corrobora a importância deste tema no campo das políticas públicas de promoção da saúde, pois, com o aumento da prevalência da hipertensão e do diabetes, medidas de controle estão sendo adotadas por diversos países.^9^ Este processo tem incentivado a utilização de diferentes intervenções, que beneficiam as condições clínicas dos pacientes, bem como possibilitam a redução dos custos para os serviços de saúde.^10^

A *World Health Organization* (WHO), motivada por países como Estados Unidos, Canadá, Austrália e Reino Unido, estimava, até o ano de 2015, reduzir em 2% ao ano as taxas de mortalidade por doenças crônicas.^11^ Seguindo estas estimativas, a OMS buscou assegurar estas ações juntamente dos governos, organizações mundiais e setores privados, aprovando o Plano de Prevenção e Controle de DCNT 2013-2020. Além disso, adotou metas voluntárias para o ano de 2025, entre elas, reduzir em 25% a mortalidade prematura por tais doenças.^1^

O crescimento do número de publicações científicas sobre as intervenções encontradas na última década pode estar relacionado ao envelhecimento populacional, pois, com o avanço da idade, as alterações fisiológicas são mais frequentes no organismo, aumentando a prevalência das DCNT.^12^ A melhora nas condições básicas de saúde, a expansão da urbanização, a comercialização global de produtos desfavoráveis a saúde e a prática de estilos de vida não saudáveis podem ser outros fatores importantes para o aumento dos estudos nesse período.^2^

Embora a hipertensão arterial seja uma doença silenciosa, que afeta indivíduos de todos os níveis socioeconômicos e apresente índices de mortalidade e prevalência global acima de outras DCNT,^5^ os números de estudos sobre intervenções associados ao paciente diabético são ainda maiores. Isto pode ser explicado pela diversidade de complicações agudas e crônicas que o *diabetes mellitus* ocasiona e, desta forma, vem trazendo ao longo dos anos, aumento de duas a três vezes nos custos de saúde em relação aos pacientes não diabéticos.^2^ Em 2017, os custos globais originados pelo diabetes foram estimados em US$ 850 bilhões, acarretando grande impacto social e econômico para os sistemas de saúde.^2^

Quanto aos tipos de intervenções que contribuem para melhorar a adesão às terapias medicamentosa e não medicamentosa, a intervenção face a face, por meio de consulta individual e visitas domiciliares, foi a mais frequente na literatura científica.^13^ As consultas individuais são muito utilizadas em assistência ambulatorial, clínicas médicas, farmácias comunitárias, equipe interdisciplinar de saúde^14^ e outros centros de saúde, visto que são métodos tradicionais em que os médicos, farmacêuticos, enfermeiros, psicólogos, nutricionistas e educadores físicos analisam, juntamente do paciente, as barreiras que impedem a adesão às terapias, apontando soluções que proporcionem melhores resultados de saúde.^15^

Em seguida, a intervenção por chamada telefônica corrobora sua eficiência, por encorajar pacientes com diversas comorbidades a adotarem melhores práticas de cuidado, por meio de ligações telefônicas para o monitoramento deles.^16^ Este tipo de intervenção é também considerado tradicional e amplamente empregado em farmácias comunitárias e clínicas pelo profissional farmacêutico, que, na função de gestor dos medicamentos, orienta os pacientes sobre comportamentos de saúde e viabiliza a melhora na adesão às terapias medicamentosa e não medicamentosa de hipertensos e diabéticos.^17^ Os serviços de ligações telefônicas proporcionam opção mais acessíveis, reduzindo a carga de consultas médicas e os custos com transportes para os pacientes de renda baixa, além de propor inserção de informações personalizadas.^18^

Na sequência, a intervenção digital, realizada por meio do envio de mensagens de texto SMS, Web, aplicativos e WhatsApp, tem mostrado, nos últimos anos, forte crescimento em estudos sobre adesão às terapias. Essas ferramentas tecnológicas vêm sendo usadas para facilitar o acesso às informações de saúde, com intuito de melhorar a qualidade de vida dos pacientes.^19^ Estudo evidenciou que o uso de diversas tecnologias pode estimular mudança nos comportamentos de saúde e intensificar a adesão às terapias de pacientes hipertensos e diabéticos.^20^

A intervenção digital tem sido eficaz principalmente em países subdesenvolvidos e em desenvolvimento, atingindo regiões de difícil acesso, assegurando que os benefícios de saúde, por meio de tecnologias estejam à disposição de toda população mundial.^21^ As ferramentas de tecnologia móvel para a saúde, conhecidas também como *m-Health,* estão sendo consideradas forte tendência para o controle de DCNT, pois apresentam baixo custo e visam à resolução de problemas de saúde à distância.^22^

Entre outras intervenções encontradas na literatura para melhorar os comportamentos de saúde em pacientes hipertensos e diabéticos, foram identificadas as intervenções indiretas, por meio de políticas públicas, diretrizes de saúde, auditorias e treinamento de profissionais.^23^

As intervenções de educação em saúde são normalmente implementadas pelos enfermeiros e outros profissionais de saúde por meio de palestras e orientações em conjunto, principalmente em comunidades em que os recursos tecnológicos são escassos ou inexistentes.^24^ Identificou-se menor quantidade de estudos, citando as intervenções por Correios, mediante o envio de cartas com recomendações de saúde^25^ e, por fim, as intervenções de incentivo financeiro, aplicadas por alguns serviços de saúde, principalmente dos setores privados, incentivando a adesão dos pacientes por compensação financeira.^26^

Em referência a origem dos estudos, a América do Norte evidenciou o maior número de pesquisas científicas relacionadas às intervenções em hipertensos e diabéticos, seguida por países do continente europeu. Nos Estados Unidos, por exemplo, um em cada três indivíduos ou 75,2 milhões de americanos têm hipertensão, sendo que quase metade desses indivíduos (35 milhões de pessoas) apresenta pressão arterial acima dos padrões recomendados.^27^ Entre os anos 2011 a 2014, o país apresentou prevalência aproximada de 34% (34,5% entre os homens e 33,4% entre as mulheres); na população idosa a prevalência foi de 67,2%, ocasionando cerca de 410.624 mortes por causa primária ou secundária, com gastos totais dado a hipertensão arterial entre 2012 a 2013 em US$ 51,2 bilhões.^28^

Já o diabetes atingiu cerca de 30,3 milhões de americanos em 2015, com prevalência de 9,4%, resultando na sétima causa de morte no país e alcançando mais de 252.806 mortes anuais por causa direta ou indireta da doença.^29^ Em 2017, os custos totais do diabetes chegaram a US$ 327 bilhões, sendo que a maior parte desses custos foram com indivíduos de faixa etária igual ou superior a 65 anos, elevando constantemente o orçamento do sistema e dos serviços de saúde.^2^ As projeções do diabetes entre a população adulta diagnosticada e não diagnosticada foram estimadas em 14% em 2010, tendendo para 21% de prevalência total até o ano 2050.^30^

A obesidade tem sido importante para o crescimento da prevalência de outras DCNT e está associada ao aumento do número de mortes prematuras por hipertensão e diabetes, principalmente em países como os Estados Unidos, que possuem alto consumo de alimentos industrializados, resultando em prevalência de 17% em crianças e um terço da população adulta aproximadamente (36,5%) com obesidade entre os anos 2011 a 2014, atingindo, em geral, indivíduos de média idade, entre 40 a 59 anos.^29^

Os países da Europa também avançaram nos estudos sobre intervenções, pois com populações envelhecidas, resultaram no crescimento permanente da prevalência de DCNT, aumentando o interesse dos países em reduzir a sobrecarga dos gastos financiados pelos sistemas de saúde.^31^

Em relação à América do Sul, composta por países em desenvolvimento, os estudos associados a intervenções para melhorar a adesão às terapias ainda são recentes e estão muito abaixo dos números norte-americanos. No Brasil, o avanço nas condições de saúde e o aumento da expectativa de vida fizeram com que o número de pessoas idosas crescesse 18% nos últimos 5 anos, saindo de 25,4 milhões no ano de 2012, para 30 milhões em 2017. Essas ocorrências, estão elevando a prevalência de DCNT, estimadas, em uma década, em um crescimento de 14,2% para a hipertensão, saindo de 22,5% no ano 2006 para 25,7% em 2016, além do diabetes, que cresceu 61,8%, passando de 5,5%, em 2006, para 8,9%, em 2016.^32^

Em 2015, as altas prevalências de DCNT ocasionaram cerca de 424.058 óbitos por doenças cardiovasculares e 62.466 mortes atribuídas ao diabetes,^33^ chegando a custos totais de US$ 4,18 bilhões e US$ 22 bilhões, respectivamente.^6 , 9^ Nos últimos anos, as mudanças provocadas no estilo de vida dos brasileiros têm influenciado o crescimento de comorbidades decorrentes da obesidade, outro importante fator de risco para a hipertensão e diabetes.^5^ A obesidade cresceu 60% no Brasil em 10 anos, partindo de 11,8%, em 2006, e chegou a 18,9%, em 2016, alcançando maior prevalência (22,9%) nos indivíduos com idade entre 55 a 64 anos.^32^

O Brasil tem adotado medidas importantes ao longo nos últimos anos para o enfrentamento das DCNT, como a implantação do Sistema de Vigilância de Doenças Crônicas Não Transmissíveis (VIGITEL), que permite o monitoramento permanente de doenças crônicas, avaliando as melhores estratégias de intervenções.^32^ Outra medida relevante foi a elaboração do Plano de Ações para o Enfrentamento de DCNT 2011-2022, que incentiva as políticas públicas de promoção da saúde, contribuindo para o cumprimento das metas, entre elas, reduzir em 2% ao ano o número de mortes prematuras por DCNT até 2022,^6^ garantindo para a saúde o desenvolvimento sustentável para a Agenda 2030.^34^

Dos estudos encontrados na literatura, aqueles que utilizaram apenas um tipo de intervenção tiveram maior frequência. Em muitos países, a gestão dos cuidados com o paciente idoso que possui duas ou mais comorbidades, torna o desafio ainda maior para gestores e profissionais de saúde. As intervenções para intensificar a adesão às terapias necessitam de diretrizes focadas em incentivos para pacientes com esta faixa etária.^35^ Nos Estados Unidos, o uso excessivo de intervenções para o mesmo paciente não tem causado melhora nos resultados de saúde, pois, além de terem características específicas para cada doença, elevam os custos, sendo necessária a busca por ferramentas de abrangência, com foco na qualidade de vida do paciente e não somente na doença.^35^

## CONCLUSÃO

O presente estudo cienciométrico apontou lacunas a serem destacadas: pequeno número de estudos realizados no continente latino-americano, especialmente no Brasil, levando em consideração o aumento da prevalência da hipertensão e do diabetes no país e em toda América Latina; apesar da evidência limitada da intervenção por incentivo financeiro, que foi utilizada apenas em alguns países desenvolvidos, a mesma merece ser destacada como uma ferramenta promissora para motivação para mudança de comportamento; e existência de poucos estudos sobre intervenções com capacidade de melhorar a adesão às terapias de pacientes que possuem comorbidades, como a hipertensão e diabetes.

Por fim, as intervenções tradicionais, como a face a face e por meio de chamada telefônica, foram as mais empregadas para melhorar a adesão às terapias anti-hipertensivas e antidiabéticas, embora o uso de tecnologias digitais emerja com tendência para promover avanços nesses comportamentos de saúde.
